# The prevalence of cardiorenal anemia syndrome among patients with heart failure and its association with all-cause hospitalizations: a retrospective single-center study from the Middle East

**DOI:** 10.3389/fcvm.2023.1244275

**Published:** 2023-09-12

**Authors:** Yosef Manla, Obada Kholoki, Feras Bader, Oshin Kanwar, Emna Abidi, Wasim S. El Nekidy, Fadi Hijazi, Nizar Attallah

**Affiliations:** ^1^ Department of Cardiology, Heart, Vascular, and Thoracic Institute, Cleveland Clinic Abu Dhabi, Abu Dhabi, United Arab Emirates; ^2^ Research Department, Academic Office, Cleveland Clinic Abu Dhabi, Abu Dhabi, United Arab Emirates; ^3^ Department of Pharmacy, Cleveland Clinic Abu Dhabi, Abu Dhabi, United Arab Emirates; ^4^ Department of Nephrology, Medical Subspecialties Institute, Cleveland Clinic Abu Dhabi, Abu Dhabi, United Arab Emirates

**Keywords:** cardiorenal anemia syndrome, cardiorenal syndrome, heart failure, Middle East, chronic kidney disease

## Abstract

**Background and aim:**

Little is known about the burden of cardiorenal syndrome (CRS) and cardiorenal anemia syndrome (CRAS) in the Middle East Region. Furthermore, whether the occurrence rates of CRAS differ across heart failure (HF) phenotypes is not widely investigated. We aimed to examine the prevalence of CRS and CRAS in patients with HF, compare characteristics of patients with CRAS-HFrEF vs. CRAS-HFpEF, and investigate anemia association with 1-year all-cause hospitalizations.

**Methods:**

HF patients who visited a multidisciplinary HF clinic at a single center between 10-2015 and 06-2022 (*n* = 968) were retrospectively included. Differences in rates of CRAS prevalence, and patients’ characteristics of those with CRAS-HFrEF vs. CRAS-HFpEF were determined using appropriate testing methods. Generalized estimating equation (GEE) models were used to determine if anemia was associated with higher rates of hospitalization.

**Results:**

CRS was prevalent in 34.4% of subjects, while 25.3% had CRAS. CRAS prevalence rates among patients with HFpEF vs. HFrEF were comparable (27.2% vs. 24.2%, *p* = 0.3). Compared to patients with HFrEF-CRAS, those with HFpEF-CRAS were more likely females (*p* < 0.001), had a higher burden of hypertension (*p* = 0.01), and lower hemoglobin (*p* = 0.02). In an adjusted GEE model, anemia was associated with an average increase of 1.8 admissions in CRS patients (*p* = 0.015).

**Conclusion:**

In patients with HF, 1 in 3 patients presented with CRS, and 1 in 4 patients had CRAS. The prevalence of CRAS was comparable among those HFpEF and HFrEF. Anemia was associated with an increased rate of 1-year all-cause hospitalization in CRS patients.

## Introduction

Cardiorenal syndrome (CRS) is a comprehensive term encompassing the intricate relationship between simultaneous cardiac and renal impairments, wherein the deterioration of one organ initiates, perpetuates, or accelerates the decline in the other (1–3). This occurs due to a vicious cycle of feedback mechanisms comprising neuroregulatory hormones, oxidative stressors, and inflammatory cytokines (3, 4). Patients with CRS may develop comorbid anemia, resulting in cardiorenal anemia syndrome (CRAS) (4). CRAS is attributed to numerous factors, including erythropoietin resistance and/or deficiency, iron deficiency, or due to the occurring inflammatory changes. As a result, patients develop impaired oxygen transport and tissue hypoxia, contributing to a worse prognosis and poor quality of life (4–7). While the current management of CRAS is multifactorial, there is a lack of specific, evidence-based recommendations for patients with this syndrome. The use of guideline-directed medical therapy for heart failure (HF) as well as erythropoietin-stimulating agents for the management of anemia in chronic kidney disease (CKD) has not been shown to be as effective in patients with CRAS as in isolated conditions (2, 6, 7). There is a need for multidisciplinary inputs to incorporate and enforce all available screening and treatment modalities.

Furthermore, heart failure with preserved ejection fraction (HFpEF) encompasses complicated pathophysiology that involves multiple systems and secondary organ dysfunction, demonstrating a considerable association between HFpEF and CRS (8, 9). One can assume a stronger tendency towards developing comorbid anemia in patients with HFpEF compared to heart failure with reduced ejection fraction (HFrEF).

There are currently limited data on the burden of CRS or CRAS in the Middle East Region. Therefore, we aimed to investigate the prevalence of CRS and CRAS in patients with HF followed in an outpatient setting, compare the characteristics of patients with CRAS-HFrEF vs. CRAS-HFpEF, and investigate the association of anemia with 1-year all-cause hospitalization in patients with CRS at a single-center in the United Arab Emirates.

## Methods

**Figure 1:**
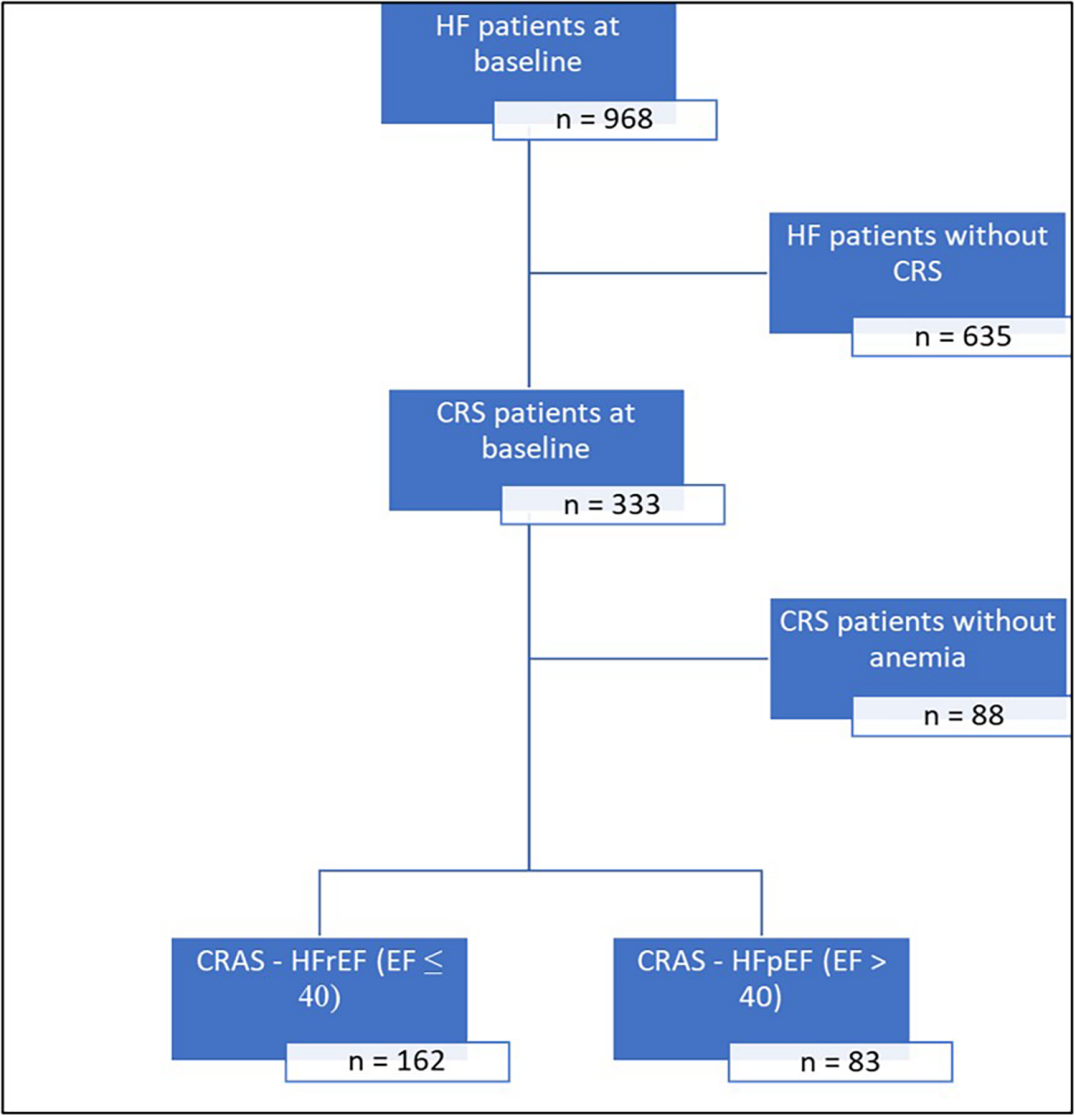
Flow diagram of patients included in this analysis. CRS, cardiorenal syndrome; CRAS, cardiorenal anemia syndrome; EF, ejection fraction; HF, heart failure; HFpEF, heart failure with preserved ejection fraction; HFrEF, heart failure with reduced ejection fraction.

Consecutive patients with chronic HF who visited a multidisciplinary HF clinic at a single center in the United Arab Emirates between October 2015 and June 2022 (*n* = 968) were retrospectively included in this analysis; Patients included in this study established care in our HF clinic following either a referral from another clinic with a diagnosis of HF based on recent HF guidelines or after an event of hospitalization for acute HF. A flow diagram of patients included in this analysis is shown in Figure 1.

Data on patient demographics, baseline comorbidities, vital signs, and laboratory findings were collected using electronic medical records. Patients were determined to have CRS (referring to type II or IV) if they were diagnosed with HF and had an (eGFR) of <60 ml/min/1.73 m^2^ calculated using the Modification of Diet in Renal Disease (MDRD) study equation (10). Patients were determined to have CRAS if they had CRS and a hemoglobin level of <130 g/L for males and <120 g/L for females. HF phenotypes were defined as having an ejection fraction (EF) of ≤40 and >40 for HFrEF and HFpEF, respectively. The study was approved by the local Research Ethics Committee, and informed consent was waived due to the retrospective nature of the study.

Descriptive statistics for categorical variables were reported as absolute numbers (%), and continuous variables were reported as a mean ± SD or median [IQR] and were compared between CRAS-HFrEF and CRAS-HFpEF groups using Chi-square test and *t*-test (or Mann-Whitney *U*-test for non-normally distributed variables) as appropriate. Unadjusted and adjusted generalized estimating equation (GEE) models were used to test the hypothesis that, among CRS patients, the presence of anemia was associated with higher counts of hospital inpatient admission. We arrived at a final model that optimized the combination of model fit and parsimony, compared to a fully adjusted GEE model containing all relevant independent variables of clinical significance (age, gender, hypertension, diabetes, hyperlipidemia, ischemic disease and baseline ejection fraction) each possible model's performance using the Quasi-Likelihood under Independence Criterion. A *p*-value <0.05 was considered to be statistically significant. All statistical analyses were performed with JMP® Data Analysis (Software Version 16, SAS Institute Inc., Cary, NC, USA), Microsoft R Open [Microsoft Corporation (2020). Microsoft R: Microsoft R umbrella package. R package version 4.0.2.] and R Studio [RStudio Team (2021). RStudio: Integrated Development Environment for R. RStudio, PBC, Boston, MA].

## Results

CRS was prevalent in 34.4% (333/968) of subjects, while 25.3% (245/968) had CRAS. Among patients with CRS, 73.6% (245/333) had CRAS (Figure 1). Among patients with CRAS, the mean age was 67.7 ± 12.6, and 33% were females. The mean hemoglobin was 105.3 ± 13.4 g/L, and the mean eGFR was 36.2 ± 14.5 ml/min/1.73 m^2^. These patients also featured a high burden of CV comorbidities, including ischemic heart disease (IHD) (62.0%), hypertension (87.8%), hyperlipidemia (77.6%), and diabetes mellitus (79.2%). The mean ejection fraction was 37.4 ± 15.86%, and the mean BMI measured 29.2 ± 7 Kg/m^2^ (Table 1).

**Table 1:** Baseline characteristics of patients with cardiorenal anemia syndrome according to their heart failure phenotype.

Variable	All (*n* = 245)	HFrEF-CRAS patients (*n* = 162)	HFpEF-CRAS patients (*n* = 83)	*p*-value
Age (years)	67.7 ± 12.6	67.6 ± 12.8	67.9 ± 12.7	0.8
Female gender	33% (81)	24.7% (40)	49.4% (41)	<0.001
Hypertension	87.8% (215)	84.0% (136)	95.2% (79)	0.01
Hyperlipidemia	77.6% (190)	75.9% (123)	80.7% (67)	0.4
Diabetes mellitus	79.2% (194)	78.4% (127)	80.7% (67)	0.7
Atrial fibrillation	31.4% (77)	29.0% (47)	36.1% (30)	0.3
History of smoking	35.9% (88)	40.7% (66)	26.5% (22)	0.03
Ischemic heart disease	62% (152)	67.9% (110)	50.6% (42)	0.01
Baseline NYHA Functional Class				
NYHA Class I	11.3%	13.9%	6.3%	0.4
NYHA Class II	50.5%	50.8%	50.0%	
NYHA Class III	35.1%	32.3%	40.6%	
NYHA Class IV	3.1%	3.1%	3.1%	
Mean ejection fraction %	37.4 ± 15.86	27.9 ± 8.1	56.1 ± 9.4	<0.001
Vitals at baseline visit				
Resting heart rate (bpm)	70.5 ± 13.1	71.4 ± 13.6	68.7 ± 12.0	0.8
Systolic blood pressure (mmHg)	125 ± 22.9	120.1 ± 20.8	133.3 ± 24.6	<0.01
Diastolic blood pressure (mmHg)	61.8 ± 13.2	61.6 ± 13.0	62.1 ± 13.6	0.5
Weight (kg)	75.7 ± 17.8	73.9 ± 17.3	79.2 ± 18.3	0.03
BMI (kg/m^2^)	29.2 ± 7	27.9 ± 6.4	31.7 ± 7.5	<0.001
Laboratory findings at baseline visit				
Hemoglobin (g/L)	105.3 ± 13.4	106.6 ± 12.7	102.8 ± 14.5	0.02
Serum creatinine (μmol/L)	157 [129–235.5]	151[129–210]	179 [129–253]	0.1
MDRD eGFR (ml/min/1.73 m^2^)	36.2 ± 14.5	37.5 ± 14.1	33.8 ± 15.0	0.1

Categorical variables were reported as absolute % (*n*), and continuous variables were reported as a mean ± SD or median [IQR]. BMI, body mass index; bpm, beats per minute; CRAS, cardiorenal anemia syndrome; HFpEF, heart failure with preserved ejection fraction; HFrEF, heart failure with reduced ejection fraction; MDRD, modification of diet in renal disease; NYHA, New York Heart Association.

Interestingly, when stratifying patients with HF according to HF phenotypes (HFrEF vs. HFpEF), rates of CRAS among patients with HFpEF vs. HFrEF were comparable (27.2% vs. 24.2%, *p* = 0.3). When comparing patient characteristics in the CRAS-HFpEF and CRAS-HFrEF groups, our study demonstrated that patients with HFpEF-CRAS were more likely females (49.4% vs. 24.7%, *p* < 0.001), had a higher burden of hypertension (95.2% vs. 84%, *p* = 0.01), and a lower burden of IHD (50.6% vs. 67.9%, *p* = 0.01). Patients with CRAS-HFpEF also had higher baseline systolic blood pressure (133.3 ± 24.6 vs. 120.1 ± 20.8 mmHg, *p* < 0.01) and higher BMI values (31.7 ± 7.5 vs. 27.9 ± 6.4 kg/m^2^, *p* < 0.001). Patients with CRAS-HFpEF also had lower hemoglobin levels (102.8 ± 14.5 vs. 106.6 ± 12.7 g/L, *p* = 0.02). There was no significant difference in the degree of renal dysfunction between both groups (*p* = 0.1).

At 1-year follow-up, 171 patients with CRS completed their follow-up visit (mean follow-up period of 369.7 ± 47.2 days). During the follow-up period, 262 events of hospitalizations occurred with an average of 1.7 and 1.1 admissions in the CRAS and non-anemic CRS patient groups, respectively. Unadjusted GEE model tested the hypothesis that the presence of anemia in CRS patients was associated with higher counts of inpatient hospitalizations. In this small sample size, the unadjusted model estimates showed an association between anemia and hospitalization that did not reach statistical significance (*β *= 0.43, *p* = 0.06). However, upon adjusting the model for patient age and the history of IHD, the model estimated that the presence of anemia was associated with an average increase of 1.8 hospital admission events (*p* = 0.015) (Table 2).

**Table 2:** Adjusted generalized estimating equations (GEE) model testing the association between the anemia and 1-year all-cause hospitalizations in patients with cardiorenal syndrome.

	*β* Estimate	Naive S.E.	Naive *z*	Robust S.E.	Robust *z*	*p* value
(Intercept)	0.003	0.236	0.011	0.207	0.013	0.99
CRAS	0.578	0.262	2.206	0.238	2.432	0.015
Age (years)	−0.001	0.009	−0.154	0.010	−0.138	0.89
Ischemic heart disease	−0.331	0.213	−1.554	0.212	−1.560	0.12

CRAS, cardiorenal anemia; S.E, standard error.

## Discussion

The present study showed that almost one-third of HF patients (34.4%) followed up at an outpatient clinic in the Middle East presented with CRS, aligning with previously published reports around the globe (1). In addition, our study revealed that 25.3% (245/968) of HF patients had CRAS, and 73.6% (245/333) of patients with CRS had concomitant anemia. Generally, the prevalence of CRAS among patients with HF ranges from 19% to 44%, while the prevalence of anemia in patients with CRS ranges from 39% to 45% (4). In a prospective, multi-center registry of 4,934 patients admitted with acute HF to 47 hospitals in seven Middle Eastern countries (Bahrain, Kuwait, Oman, Qatar, Saudi Arabia, United Arab Emirates, and Yemen), CRAS was evident in 27% of the cases (6). This is comparable to the findings of our study despite it being conducted in outpatient settings.

In our paper, we aimed to explore the association between CRAS and HF phenotypes. However, our results showed no difference in the prevalence of CRAS among patients with HFpEF vs. HFrEF, despite HFpEF representing a more complicated and systematic syndrome. We found that patients with CRAS-HFpEF were more likely females, obese, and had hypertension, in accordance with the typical clinical picture of HFpEF patients (8). In addition, patients with HFpEF CRAS had lower hemoglobin, highlighting the profound effect of undergoing mechanisms in HFpEF on developing anemia (11). Patients in advanced stages of HF and CKD manifest anemia more severely (12).

Silverberg et al. showcased the positive correlation between worsening cardiovascular and renal function on the development of anemia, causing up to 70% and 49% increase in the prevalence of anemia, respectively (12).

Our study also evaluated the association of having anemia with the incidence of 1-year all-cause hospitalization in CRS patients, and we found that patients with CRAS were hospitalized an additional 1.8 times, on average, after adjusting for age and history of IHD. Previous studies have strongly linked anemia with poor outcomes and quality of life. Al-Jarallah et al. reported in their multi-center study from the Middle East that patients with CRAS had higher odds of all-cause mortality during hospital admission [adjusted odds ratio (aOR), 2.10; 95% confidence interval (CI): 1.34–3.31], and at 12 months follow-up (aOR, 1.45; 95% CI: [1.12–1.87]) (6). Another study conducted by Kim et al. showed that patients with CRAS were more likely to develop non-fatal myocardial infarctions, be re-hospitalized due to HF exacerbations, and die from cardiovascular causes compared to patients without CRAS (7). While hospitalized, patients with such a morbidity profile should benefit from multidisciplinary interventions during hospitalization, which can further improve patient outcomes, including reducing rehospitalization rates (13). Moreover, we noticed that the number of patients with CRS evaluated at 1-year follow-up was significantly lower than those seen at initial visits. This loss of patient follow-up might be attributed to visiting multiple providers and patient non-adherence, highlighting the need for multidisciplinary cardio-renal metabolic clinics that address these comorbidities at once (14, 15).

Our study had several limitations. This is a retrospective analysis of patients' baseline kidney function and hemoglobin at their first visit to the HF clinic, and being a single-point estimation of eGFR might underestimate the burden of CRAS as it fluctuates due to many factors (e.g., Medication). In addition, given the high prevalence of DM in our region (16), the resultant hyperfiltration in the early stage of diabetic nephropathy might have masked the worsening in renal function. Furthermore, GFR was estimated using the MDRD equation, which is less accurate at higher GFR values (10). Also, the presence of dilutional anemia among patients with volume overload might overestimate the prevalence of CRAS. Moreover, being a single-center study might limit the study's external validity, which calls for multi-center studies to evaluate the true burden of the disease. Lastly, we did not evaluate other clinical outcomes, such as mortality or quality of life measures, due to the study's retrospective nature and the study's sample size.

## Conclusion

In this single-center study, approximately 1 in 3 patients with HF presented with CRS, and 1 in 4 patients with HF had CRAS. Interestingly, the prevalence of CRAS was comparable between HFpEF and HFrEF patients. In addition, anemia was associated with an increased rate of 1-year all-cause hospitalization in patients with CRS. Future multi-center studies are warranted to better estimate and tackle the burden of CRAS in the region.

## Data Availability

The raw data supporting the conclusions of this article will be made available by the authors, without undue reservation.
